# Exploring Diagnostic Classification Prevalence in German and Dutch Child and Adolescent Psychiatry: An International Cross-Sectional Comparative Study

**DOI:** 10.1177/13634615261428852

**Published:** 2026-04-10

**Authors:** Bas T. H. de Veen, Pierre C. M. Herpers, Martine van Dongen-Boomsma, Volker Reissner, Martin Knollmann, Ingo Spitczok von Brisinski, Johannes Hebebrand, Wouter G. Staal, Ron H. J. Scholte

**Affiliations:** 1 159214 Radboud University, Behavioural Science Institute, Nijmegen, Netherlands; 2 Karakter, Child and Adolescent Psychiatry, Nijmegen, Netherlands; 3 1787 Center of Expertise for Youth Depression, GGZ Oost Brabant, Oss, Netherlands; 4 Department of Child and Adolescent Psychiatry, Psychosomatics and Psychotherapy, 27170 University of Duisburg-Essen, University Hospital Essen, Essen, Germany; 5 62615 LVR-Klinik Viersen, Child and Adolescent Psychiatry, Viersen, Germany; 6 Department of Psychiatry, Radboud University Medical Centre, Nijmegen, The Netherlands; 7 Leiden Institution for Brain and Cognition, Leiden University, Leiden, The Netherlands

**Keywords:** child and adolescent psychiatry, classification, prevalence, international, mental disorders

## Abstract

Classification systems like the DSM-5 and ICD-10 facilitate international comparative research on mental disorders. However, few studies have compared classification distributions in child and adolescent psychiatric settings across countries. This study explored similarities and differences in classification prevalence between German and Dutch children and adolescents referred to psychiatric facilities. Data were retrospectively collected from clinical samples of inpatients and outpatients who underwent diagnostic assessments in Germany (*n* = 7,089) and the Netherlands (*n* = 2,574), aged 0–18 years (*M* = 12.70; *SD* = 3.82). A multivariate analysis compared primary classifications between the two samples, which were further stratified into three age groups: early childhood (0–5 years), middle childhood (6–12 years), adolescence (13–18 years). The main factor influencing classification was the country. Age and sex showed moderate to low effect sizes, respectively. The impact varied across different age groups and sexes. Patients in the German sample were generally older and had a higher proportion of girls than those in the Dutch sample. Mood, anxiety, disruptive/impulsive-control and conduct, and trauma and stressor-related disorders were more prevalent in the German sample, while autism spectrum disorder and attention-deficit/hyperactivity disorder were more common in the Dutch sample. Our findings suggest that the primary classifications of mental disorders in Dutch and German children and adolescents largely depend on the country. This may have implications for cross-country comparisons and highlights the potential influence of national mental healthcare systems and cultural contexts on classification practices, which could impact policy decisions.

## Introduction

The Diagnostic and Statistical Manual of Mental Disorders (DSM; American Psychiatric Association) and the International Classification of Diseases (ICD; World Health Organization) are internationally recognized classification systems widely used for clinical, research and policy purposes in child and adolescent psychiatry (CAP) (American Psychiatric Association, 2013; World Health Organization, 1992). While both systems aim to facilitate global comparability, their adoption across countries may vary due to contextual factors. These can be understood as broader environmental, societal, and systemic influences, including historical, economic and healthcare system characteristics that are permeated by cultural norms and values. However, the specific reasons why countries adopt one classification system over the other are not well delineated in the international literature. For clinicians, classifications are useful tools because they provide a standardized vocabulary for describing symptoms and defining disorders. As such, they support clinicians to communicate about a diagnosis with the patient, their parents and colleagues through a standardized clustering and naming of symptoms. Classification systems have also enabled care organization to centralize around specific classifications (e.g., specialized clinical units for treatment of depression, etc.). Furthermore, these classification systems enable researchers to systematically categorize and study psychiatric conditions, aiding in understanding the underlying causal mechanisms and therapeutic possibilities regarding these conditions. So, DSM and ICD have been adopted in countries across the world to provide structure, facilitate organization and support investigation in mental healthcare. Therefore, it is particularly important for clinicians, researchers, and policymakers not only to be aware of the benefits of these classification systems but also of the limitations of these models in classifying psychiatric disorders (Jablensky, 2016; Kapadia et al., 2020). Potential limitations, such as the validity and utility of these classification systems have also been debated, questioning the value of categorical versus dimensional approaches, cultural sensitivity, reliability and consistency of diagnoses, and the risk of overdiagnosis of mental disorders (Angold & Jane Costello, 2009; de Ridder & van Hulst, 2023; Lord et al., 2012; Merten et al., 2017). In addition, concerns have been voiced about the comparability of classification outcomes across different sites and national contexts (Canino et al., 1997; Lord et al., 2012).

Despite the aim of both the DSM and ICD classification systems to facilitate internationally applicable frameworks for comparative research, cultural or organizational variations between countries may hamper reliable interpretation and application of these systems. Studies on the prevalence of classifications between countries mainly focused on the general population (Baumeister & Härter, 2007; Heim et al., 2017; Merikangas et al., 2009; Polanczyk et al., 2015). A systematic review study by Polanczyk et al. (2015) found a worldwide pooled prevalence of mental disorders among children and adolescents of 13.4%. Furthermore, a different study on diagnosis prevalence in clinical settings identified oppositional defiant disorder and attention deficit/hyperactivity disorder (ADHD) as the most common psychiatric diagnoses (Hardan & Sahl, 1997). Yet, to the best of our knowledge, cross-country comparative research on classification distributions in CAP does not exist. As such, our understanding of the classification in CAP across different national contexts remains largely incomplete. Identifying classification distributions could elucidate potential differences in prevalence between countries, which may be influenced by multiple factors, including societal and cultural values and norms, child rearing and didactic strategies, and economic conditions. Furthermore, healthcare characteristics, including institutional embedding, funding structures, reimbursement policies, referral pathways, accessibility of care, quality and coverage of care services such as service-specific diagnostic culture and conventions, and country-specific theoretical frameworks in psychiatry may influence classification patterns in child and adolescent psychiatry. For example, care for specific disorders such as autism spectrum disorder (ASD) is provided primarily by specialised autism clinics in Germany but mainly within the general child and adolescent psychiatry in the Netherlands. Consequently, Dutch child and adolescent psychiatric caregivers overall may develop more diagnostic focus on this disorder than the general German clinical practice. The present study is a first step to explore whether differences in classifications in child and adolescent psychiatry exist between countries, providing a foundation for future research into the factors shaping these discrepancies.

This retrospective, cross-sectional comparative study explores classification distributions in two adjacent countries that have comparable (genetic) populations and socio-economic conditions. Specifically, it compares diagnostic classification among children and adolescents with mental health problems receiving CAP services between Germany and the Netherlands, correcting for age and sex. The findings may offer new insights into classification practices of childhood psychiatric disorders in distinct national contexts and contribute to a better understanding of the consistency and comparability of psychiatric disorder classifications across countries.

## Method

### Sample

A total of 9,663 children and adolescents (3,925 girls, 40.6%), with a mean age of 12.70 years (*SD* = 3.82; range 0–18 years), were collected from patient records from the year 2017. The Dutch sample contained 2,574 (26.6%) participants and the German sample consisted of 7,089 (73.4%) participants. The total study sample contained both inpatients and outpatients. All patient records were anonymized and both samples in this study consisted of children and adolescents who underwent diagnostic assessments.

Children and adolescents were included based on the criteria of having received an intake. In cases of multiple intakes in the same year, only the most recent assessment was included. Participants were referred to child and adolescent psychiatric clinics, two in Germany, (Landschaftsverband Rheinland [LVR]) clinic Essen and Viersen, and one located in the Netherlands (Karakter, Child and Adolescent Psychiatry at Nijmegen). Based on general referral data from the Dutch clinic, the majority of patients are mainly referred by general practitioners, with smaller contributions from medical specialists and municipalities. In Germany, patients are generally referred by outpatients clinics of general practitioners, paediatricians, and child and adolescent psychiatrists. In German and Dutch psychiatry the upper age limit is 18 years. Adolescents of 18 years and above were typically transferred to adult mental health services. Given the study focused on children and adolescents, all cases aged 18 years and older were excluded.

### Measures

Information on age, sex, primary classifications was obtained from patient records. The age of the children was based on the registration date and rounded down to a whole number and categorized into early childhood (0–5 years), middle childhood (6–12 years), adolescence (13–18 years) in order to assess age-group specific differences.

In clinical routine, a multidisciplinary team of experts determines clinical classifications, that is, a child psychiatrist and psychologist collaborated to reach a consensus diagnosis by assessing the child's developmental history, conducting observations, performing a psychiatric evaluation, and reviewing teacher reports and documentation from other professionals involved in the child's care. Clinical diagnoses were assigned according to the ICD-10 (World Health Organization, 1992) (German clinics) or DSM-5 classification (American Psychiatric Association, 2013) (Dutch clinic). A cross-system mapping of classification distributions (i.e., “crosswalk”) was performed to align and compare broad diagnostic categories between DSM-5 and ICD-10 primary classifications (American Psychiatric Association, 2013; Dilling & Reinhardt, 2015; San Francisco Health Network, 2015). Classifications were mapped from DSM-5 to ICD-10 codes. The mapping was implemented using SPSS syntax and was manually reviewed by a child and adolescent psychiatrist to ensure accuracy. Primary classifications were grouped to include the most prevalent used subcategories of mental, behavioural and neurodevelopmental disorders with a prevalence of > 5% in the total sample. In the case of multiple diagnoses in the same year, only the most recent diagnostic assessment outcomes were included.

### Statistical Analyses

All analyses were performed using SPSS version 27. The assumption of normality was assessed through visual inspection. Descriptive statistics were calculated for age (years), sex (percentage female), and distribution of diagnostic classifications (frequency and percentage per category). A Multivariate Analysis of Covariance (MANCOVA) was used to assess the impact of country on diagnostic classifications, controlling for age and sex. To further explore the significant multivariate effects found in the MANCOVA, post hoc analyses were conducted. Chi-square (*χ^2^*) tests were used for categorical variables and independent sample *t*-tests were used to compare continuous variables between the two samples.

## Results

### Multivariate Analysis of Classification Differences

The effect of country on primary classifications was statistically significant (*Wilks’ λ* = 0.140; *F* = 2,458.42; *p* < .001) while controlling for age, sex and interaction effects (Table 1). This analysis showed that there were substantial differences in primary classifications between German and Dutch samples suggesting classifications are assigned differently depending on the country. The effect showed a large effect size (*η^2^_p_* = .860), indicating 86.0% of the variance can be explained by the country variable. Both age and sex had significant effects on primary classifications between German and Dutch samples, suggesting classifications were also assigned differently depending on the age and sex of children and adolescents. While the effect of age was statistically significant (*Wilks’ λ* = 0.852; *F* = 69.55; *p* < .001), the associated effect showed a moderate effect size (*η^2^_p_* = .148). Similarly, the effect of sex was statistically significant (*Wilks’ λ* = 0.902; *F* = 43.64; *p* < .001), also with a moderate effect size (*η^2^_p_* = .098). Furthermore, interaction effects between country, age and sex on primary classifications were also statistically significant. The interaction between country and age was significant (*Wilks’ λ* = 0.889; *F* = 50.15; *p* < .001), with a moderate effect size (*η^2^_p_* = .111) indicating classifications in countries may vary across different age groups. Similarly, the interaction between country and sex also reached statistical significance (*Wilks’ λ* = 0.961; *F* = 16.10; *p* < .001), although with a small effect size (*η^2^_p_* = .039), suggesting that the impact of country on classifications is not consistent between sexes. Follow up analyses were conducted to compare specific classifications between the countries for different age groups and sex.

**Table 1.** Comparison of Age, Sex and Primary Classifications.

	Comparison of Children and Adolescents from German vs. Dutch Sample				
	Multivariate Analysis				
	*Wilks’ λ*	*df*	*F*	*p*	*η^2^_p_*
*Main effect*					
Country	0.140	24	2,458.42	<.001	.860
*Covariates*					
Age	0.852	24	69.55	<.001	.148
Sex	0.902	24	43.64	<.001	.098
*Interaction effects*					
Country × age	0.889	24	50.15	<.001	.111
Country × sex	0.961	24	16.10	<.001	.039

*Note.* Table 1 presents a multivariate analysis on the comparison of children and adolescents from German vs. Dutch samples. Group differences were assessed using *Wilks’ λ* in the context of the MANCOVA. A lower *Wilks’ λ* value indicates more substantial differences between groups in terms of classification variations. A higher *F* value indicate a stronger effect of the independent variable on these variations. The effect size measure *η^2^_p_* quantifies the proportion of variance attributable to the independent variables, with larger values indicating greater impact. Statistical significance is indicated by *p-value*.

### Post Hoc Analyses by Age Group and sex

#### Age Group Characteristics

Overall, the composition of the German and Dutch samples varied significantly across three different age groups (Table 2; *χ^2^* = 358.62; *df* = 2; *p* < .001): early childhood, middle childhood, and adolescence. Notably, the mean age of the total German sample (*M* = 13.07; *SD* = 3.63) was higher than that of the Dutch sample (*M* = 11.67; *SD* = 4.13) (*F* = 87.17; *p* < .001). While the mean age of early childhood and middle childhood was comparable between the German and Dutch samples, the mean age of adolescents differed significantly between the two countries (*F* *=* 0.042; *p* = .01), likely due to the large sample size. The sex ratio varied between the two countries for the total sample, with 42.9% girls in the German sample and 34.4% in the Dutch sample (*χ^2^* = 55.88; *p* < .001). In the adolescent group, the German sample had significantly more girls (52.0%) than the Dutch sample (42.2%) (*χ^2^* = 33.06; *p* < .001).

**Table 2.** Classifications of German and Dutch Samples per Age Category.

	Early Childhood (0–5 years)		Middle Childhood (6–12 years)		Adolescence (13–18 years)		Total (0–18 years)	
	Germany	Netherlands	Germany	Netherlands	Germany	Netherlands	Germany	Netherlands
% of total respective sample (*n*)	2.0 (139)^x^	9.3 (240) ^x^	38.4 (2,725) ^x^	45.7 (1,177) ^x^	59.6 (4,225) ^x^	44.9 (1,157) ^x^	100 (7,089)	100 (2,574)
Age	4.2 ± 1.0	4.1 ± 1.0	9.5 ± 1.9	9.4 ± 1.9	15.6 ± 1.6*	15.5 ± 1.6*	13.1 ± 3.6*	11.7 ± 4.1*
Sex								
Girls	31.7 (44)	25.0 (60)	29.3 (799)	28.5 (335)	52.0 (2,196)*	42.4 (491)*	42.9 (3,039)*	34.4 (886)*
Boys	68.3 (95)	75.0 (180)	70.7 (1,926)	71.5 (842)	48.0 (2,029)*	57.6 (666)*	57.1 (4,050)*	65.6 (1,688)*
Primary classifications								
ASD	25.9 (36)*	55.0 (132)*	9.9 (271)*	38.2 (450)*	5.2 (218)*	37.3 (431)*	7.4 (525)*	39.4 (1,013)*
ADHD	12.9 (18)	10.0 (24)	31.5 (858)*	40.6 (478)*	9.4 (398)*	22.2 (257)*	18.0 (1,274)*	29.5 (759)*
Neurodevelopmental disorders other specified	15.1 (21)	19.6 (47)	8.8 (239)*	5.9 (69)*	3.1 (131)	2.3 (27)	5.5 (391)	5.6 (143)
MD	0.0 (0)	0.4 (1)	1.9 (51)	1.4 (16)	23.1 (975)*	9.6 (111)*	14.5 (1,026)*	5.0 (128)*
AD	7.2 (10)	3.8 (9)	11.6 (316)*	4.3 (51)*	10.6 (447)*	6.7 (77)*	10.9 (773)*	5.3 (137)*
DICCDs	7.2 (10)	3.3 (8)	17.5 (477)*	3.0 (35)*	11.9 (501)*	4.9 (57)*	13.9 (988)*	3.9 (100)*
TSRD	19.4 (27)*	2.1 (5)*	11.3 (307)*	3.4 (40)*	15.4 (652)*	7.7 (89)*	13.9 (986)*	5.2 (134)*
Other disorders	12.2 (17)	5.8 (14)	6.3 (172)	3.2 (38)	19.8 (836)	9.3 (108)	15.9 (1,126)	6.2 (160)
Missing / no diagnosis	0.0 (0)	0.0 (0)	1.2 (34)	0.0 (0)	1.2 (67)	0.0 (0)	1.4 (101)	0.0 (0)

*Note.* Table 2 presents descriptive statistics for the samples per age group, expressed as % within country (*n*) or *Mean* ± *SD*. Group differences were assessed using *χ^2^* and independent samples tests. *p* < .05 is denoted by an asterisk (*) within each row. The lower case “x” indicates a statistically significant (*p* < .05*)* difference in proportion between age groups. Statistically significant variables are shown in bold. Only diagnostic classifications with > 5% prevalence in the total sample are included. Abbreviations: ASD = autism spectrum disorder; ADHD = attention-deficit/hyperactivity disorder; MD = mood disorders; AD = anxiety disorders; DICCDs = disruptive, impulsive-control and conduct disorders; TSRD = trauma and stressor-related disorders.

#### Classification Differences by Age Group

Differences in distributions by age groups were analysed across the most prevalent classifications and compared among German and Dutch samples. In addition, the prevalence within each age group was analysed separately for the German and Dutch samples for each classification.

The total German sample exhibited higher prevalence rates for mood disorders (MD) (14.5% vs. 5.0%; *χ^2^* = 162.07; *df* = 1; *p* < .001), anxiety disorders (AD) (10.9% vs. 5.3%; *χ^2^* = 68.97; *df* = 1; *p* < .001), disruptive, impulsive-control and conduct disorders (DICCDs) (13.9% vs. 3.9%; *χ^2^* = 190.97; *df* = 1; *p* < .001), and TSRD (13.9% vs. 5.2%; *χ^2^* = 139.58; *df* = 1; *p* < .001). Specifically, MD were more prevalent among German adolescents (23.1% vs. 9.6%; *χ^2^* = 102.52; *df* = 1; *p* < .001) than among their Dutch peers. Moreover, German children in middle childhood (11.6% vs. 4.3%; *χ^2^* = 50.89; *df* = 1; *p* < .001) and adolescents (10.6% vs. 6.7%; *χ^2^* = 15.92; *df* = 1; *p* < .001) exhibited significantly higher prevalence rates of AD than their Dutch counterparts. Similarly, DICCDs were notably more prevalent in the German sample than in the Dutch sample in the middle childhood (17.5% vs. 3.0%; *χ^2^* = 152.25; *df* = 1; *p* < .001) and adolescence age groups (11.9% vs. 4.9%; *χ^2^* = 46.96; *df* = 1; *p* < .001). TSRD were markedly more prevalent in the German sample across all age groups: early childhood (19.4% vs. 2.1%; *χ^2^* = 34.24; *df* = 1; *p* < .001), middle childhood (11.3% vs. 3.4%; *χ^2^* = 62.80; *df* = 1; *p* < .001), and adolescence (15.4% vs. 7.7%; *χ^2^* = 45.83; *df* = 1; *p* < .001). While the classification “Neurodevelopmental disorders other specified” did not vary in the total sample, this classification was more prevalent in the German sample among children in the middle childhood group (8.8% vs. 5.9%; *χ^2^* = 9.56; *df* = 1; *p* = .002).

The total German sample exhibited significantly lower prevalence rates of ASD (7.4% vs. 39.4%; *χ^2^* = 1,440.28; *df* = 1; *p* < .001) and ADHD (18.0% vs. 29.5%; *χ^2^* = 150.74; *df* = 1; *p* < .001) compared to the Dutch sample. Notably, ASD was significantly less prevalent in the German sample across all age groups: early childhood (25.9% vs. 55.0%; *χ^2^* = 30.21; *df* = 1; *p* < .001), middle childhood (9.9% vs. 38.2%; *χ^2^* = 436.65; *df* = 1; *p* < .001), and adolescence (5.2% vs. 37.3%; *χ^2^* = 882.08; *df* = 1; *p* < .001). Similarly, ADHD was less prevalent in the German sample for the middle childhood (31.5% vs. 40.6%; *χ^2^* = 30.40; *df* = 1; *p* < .001) and adolescence (9.4% vs. 22.2%; *χ^2^* = 139.06; *df* = 1; *p* < .001).

#### Classification Differences by Sex

Differences in sex distributions were analysed across the most prevalent classifications and compared among German and Dutch samples. In addition, the proportion of girls’ and boys’ prevalence in the German and Dutch sample were analysed for each classification.

Prevalence rates among girls were significantly higher in the German sample compared to the Dutch sample for MD (Table 3; 24.0% vs. 9.7%; *χ^2^* = 84.73; *df* = 1; *p* < .001), AD (14.0% vs. 9.8%; *χ^2^* = 10.64; *df* = 1; *p* < .001), DICCDs (12.3% vs. 3.5%; *χ^2^* = 57.21; *df* = 1; *p* < .001), and TSRD (16.3% vs. 7.8%; *χ^2^* = 40.29; *df* = 1; *p* < .001). Similarly, prevalence rates of boys were also significantly higher in the German sample compared to the Dutch sample for MD (7.4% vs. 2.5%; *χ^2^* = 50.70; *df* = 1; *p* < .001), AD (8.6% vs. 3.0%; *χ^2^* = 58.14; *df* = 1; *p* < .001), DICCDs (15.2% vs. 4.1%; *χ^2^* = 139.75; *df* = 1; *p* < .001), and TSRD (12.1% vs. 3.9%; *χ^2^* = 93.18; *df* = 1; *p* < .001).

**Table 3.** Classifications of German and Dutch Samples per Sex.

	Classification Distribution per Sex			
	Girls		Boys	
	Germany	Netherlands	Germany	Netherlands
Primary classifications				
ASD	2.9 (88)*	26.6 (236)*	10.8 (437)*	46.0 (777)*
ADHD	7.1 (215)*	25.8 (229)*	26.1 (1,059)*	31.4 (530)*
Neurodevelopmental disorders other specified	3.9 (118)*	5.5 (49)*	6.7 (273)	5.6 (94)
MD	24.0 (728)*	9.7 (86)*	7.4 (298)*	2.5 (42)*
AD	14.0 (426)*	9.8 (87)*	8.6 (347)*	3.0 (50)*
DICCDs	12.3 (373)*	3.5 (31)*	15.2 (615)*	4.1 (69)*
TSRD	16.3 (495)*	7.8 (69)*	12.1 (491)*	3.9 (65)*
Other disorders	17.7 (538)	11.2 (99)	12.0 (487)	3.6 (61)
Missing / no diagnosis	1.9 (58)	0.0 (0)	1.1 (43)	0.0 (0)

*Note.* Table 3 presents descriptive statistics for the samples per sex, expressed as % within country (*n*). Group differences were assessed using *χ^2^*. *p* < .05 is denoted by an asterisk (*) within each row and statistically significant variables are shown in bold. Only diagnostic classifications with > 5% prevalence in the total sample are included. Abbreviations: ASD = autism spectrum disorder; ADHD = attention-deficit/hyperactivity disorder; MD = mood disorders; AD = anxiety disorders; DICCDs = disruptive, impulsive-control and conduct disorders; TSRD = trauma and stressor-related disorders.

In contrast, the German sample showed significantly lower prevalence rates among girls than the Dutch sample for ASD (2.9% vs. 26.6%; *χ^2^* = 1,440.28; *df* = 1; *p* < .001) and ADHD (7.1% vs. 25.8%; *χ^2^* = 150.74; *df* = 1; *p* < .001), and neurodevelopmental disorders other specified (3.9% vs. 5.5%; *χ^2^* = 150.74; *df* = 1; *p* < .001). Similarly, the German sample showed a similar pattern for boys prevalence rates, with significantly fewer boys being classified with ASD (10.8% vs. 46.0%; *χ^2^* = 887.02; *df* = 1; *p* < .001) and ADHD (26.1% vs. 31.4%; *χ^2^* = 16.40; *df* = 1; *p* < .001) compared to the Dutch sample.

## Discussion

This retrospective, cross-sectional comparative study explored the similarities and differences in the prevalence of mental health classifications in a clinical sample of children and adolescents with mental health problems in German and Dutch child and adolescent psychiatry. In a German-Dutch cross-border region with a predominantly white population in Western Europe, significant differences in psychiatric classifications were found between Germany and the Netherlands. Across both countries, anxiety disorders and mood disorders were more prevalent from middle childhood onwards, and disruptive, impulse-control, and conduct disorders showed a similar pattern, particularly in Germany. German children were more likely to have a classification of mood disorders, anxiety disorders, disruptive, impulsive-control and conduct disorders, and trauma and stressor-related disorders. In contrast, Dutch children were more likely to have a classification of autism spectrum disorder and ADHD.

Our findings regarding higher prevalence of anxiety disorders from middle childhood onwards are consistent with a meta-analysis on the median age of onset of specific mental disorders worldwide (Solmi et al., 2022). That study showed that these disorders typically emerge during middle childhood and adolescence. The similar prevalence rates between the German and Dutch samples may be attributable to the unique challenges of clinical assessments in infants and toddlers, including the heterogenous symptom presentation and uncertainty about the developmental significance of early clinical symptoms (Drotar, 2002; Frankel et al., 2004). These challenges may contribute to delayed or cautious diagnostic decision-making, resulting in children receiving classifications at a later age. In addition, the cognitive, social-emotional development of these phases may also prevent some of these disorders. Finally, our study found lower prevalence rates of autism spectrum disorder in the German sample and a higher prevalence of trauma and stressor-related disorders than in the Dutch sample across all ages. Despite the distinct origins of these disorders, their occurrence and symptoms may overlap as individuals with autism spectrum disorder may be more vulnerable to victimization and coping with traumatic events (Haruvi-Lamdan et al., 2018; Kerns et al., 2015; Rosen et al., 2018). Hence, these findings underscore the need for more research on the question why prevalence rates for autism spectrum disorder and trauma-related disorders differ consistently between German and Dutch clinics across all ages.

The present study found significant cross-national differences in the prevalence of autism spectrum disorder, attention-deficit/hyperactivity disorder, mood disorders, anxiety disorders, disruptive, impulse-control, and conduct disorders, and trauma- and stressor-related disorders when comparing German and Dutch boys and when comparing German and Dutch girls. Cultural, alongside other contextual factors, may contribute to cross-national variations. These factors may also have contributed to the prevalence variations between German and Dutch boys and between German and Dutch girls (Altemus et al., 2014; Begeer et al., 2013; Carter et al., 2011; Lewinsohn et al., 1998; Mowlem et al., 2019). For example, cultural gender stereotypes related to masculinity and femininity have been shown to influence anxiety levels (Carter et al., 2011). Specifically, Carter et al. (2011) found that youths with more advanced pubertal development and high levels of femininity reported stronger anxiety symptoms, whereas those with high levels of masculinity had lower anxiety levels (Carter et al., 2011). Consequently, cultural differences in gender roles and behavioural stereotypes related to femininity and masculinity may influence the prevalence rates of anxiety and depression between countries (Huang et al., 2016; Marques et al., 2011; Nyroos et al., 2015). Altogether, our findings underscore the need to further investigate the moderating role of country and sex-specific nuances on classification prevalence rates.

Although the influence of contextual factors, and more specifically healthcare system characteristics, was outside the scope of this study, several factors could nonetheless influence the observed differences in classification. These factors may include differences in training of mental health professionals, variations in reimbursement policies, the use of different classification systems, divergent referral pathways and early-detection practices, organizational emphasis on particular mental health problems, difference in treatment duration and modality, and differences in roles of professionals, such as psychiatrists, and psychologists (Evans et al., 2013; First et al., 2021; Heim et al., 2019; Hofstede, 2011; Lord et al., 2012; Malich, 2020; Marques et al., 2011). Furthermore, it can be assumed that the differences observed in this study might be attributable to rather subtle influences. These may include specific factors such as attitudes of healthcare professionals or public awareness and understanding of certain diagnoses. For example, differences could arise from varying levels of public interest in autism, the acceptance of autism as an explanation for problems and symptoms that may have other causes, or differing degrees of scepticism or openness towards autism as a spectrum disorder. Additionally, healthcare professionals may hold different views on how broadly the autism spectrum should be defined. While these factors may contribute to cross-national differences in classification prevalence, delineating their individual impact is challenging and lies outside the scope of this study. To better understand these mechanisms, further research is needed.

Cultural factors may also influence classification prevalence through differences in social norms, interpretations of psychological distress, help-seeking behaviour, patient-clinician communication, symptom presentation, and clinical perceptions (Biswas et al., 2016; Compton & Shim, 2015; Kirmayer, 2001; Kirmayer & Ryder, 2016; Manago, 2015; Meeuwesen et al., 2009; Sileo & Kershaw, 2020). Although the Netherlands and Germany are neighbouring countries with broadly similar Western European cultural backgrounds, research suggests that cultural differences exist (Hofstede, 2011). For example, according to this model, German society scores higher than Dutch society on the masculinity scale, indicating a stronger separation of emotional roles between men and women in Germany. Furthermore, German society scored lower on the indulgence scale compared to Dutch society, suggesting greater restraint in the gratification of natural human desires related to enjoying life. These differences may affect the diagnostic process and ultimately classification practices. Research indicates that cultural context may also influence how symptoms are expressed and interpreted (Biswas et al., 2016; Kirmayer, 2001; Kirmayer & Ryder, 2016). For example, several studies found that cultural contexts may influence how symptoms of anxiety, depression and other disorders are presented (Kirmayer, 2001; Kirmayer & Ryder, 2016). Likewise, Biswas et al. (2016) showed that therapists from different cultural backgrounds assessed the importance of symptoms differently, suggesting that clinical evaluation may be influenced by cultural factors (Biswas et al., 2016). Hence, such influences may affect reported prevalence of classifications (Canino & Alegría, 2008).

Given the close geographic proximity of participants in the cross-border region, biological and genetic variations are unlikely to be a major factor for the observed differences (Lao et al., 2008). Instead, contextual (e.g., environmental) factors may play a more significant role. Bronfenbrenner (1994) already emphasized that human development stems from the interplay between individual attributes and contextual factors over time (Bronfenbrenner, 1994). Consequently, mental health problems may arise from a combination of the lack of protective factors and the presence of negative dynamics shaped by influences such as healthcare system characteristics and cultural influences (Rutter, 2012). National cultural differences between Germany and the Netherlands may be less pronounced in cross-border regions because of sustained interaction and daily mobility across the border. While the two healthcare systems differ, both countries offer highly developed services and possess extensive child and adolescent psychiatric expertise. Although our study did not assess cultural or healthcare variables, such influences could in part explain the classification differences observed in this study. To the best of our knowledge, no previous research has examined these factors in this specific setting. Follow-up studies are clearly needed to identify which cultural or care system-level factors shape classification patters, and in turn, how they might affect care quality.

Several limitations should be mentioned. First, this study compared classification distributions between different classification systems using a DSM-ICD crosswalk, which may have affected classification prevalence (American Psychiatric Association, 2013; Doernberg & Hollander, 2016). However, since the primary focus of the study was to assess general patterns rather than conduct an in-depth analysis of diagnostic specifics, minor variations introduced by the crosswalk are unlikely to have significantly impacted the study's findings and conclusions. Second, we used only primary classification distributions, excluding secondary and other classifications. While including these might have provided a more comprehensive overview of classifications, primary classifications were chosen due to their relevance to the patient's major complaints or most severe mental health problems. Lastly, this study compared only German and Dutch child and adolescent psychiatric samples, making it difficult to generalize our findings to other countries and organizations. Despite this limitation, the study's value lies in its contribution to understanding classification patterns within these contexts. As such, it provides a foundation for broader future research that could expand to other countries and additional organizations.

## Conclusion

This study found that significant differences in classification distributions may exist between child and adolescent psychiatric clinics in Germany and the Netherlands. These findings suggest that such differences may be due to variation in national classification practices. The insights derived from this study may be relevant not only for clinical decision-making, but also for future research and policy development. Additionally, it is important to explore how cultural norms may shape the organisation of care systems and may influence the accessibility and quality of mental health services for children and adolescents. Future research is needed to further investigate the interplay between cultural and contextual factors, and classification practices across national systems.
